# Correspondence

**Published:** 1907-07

**Authors:** James B. Baird

**Affiliations:** S. S. Prinzess Alice, North German Lloyd Line, Midocean


### CORRESPONDENCE

S. S. Prinzess Alice,
North German Lloyd Line,
Midocean, May 30, 1907.

Editor Atlanta Journal-Record of Medicine—

Dear Sir:

A great ocean steamship constructed, equipped and managed on modern lines is a veritable floating city, equaling or surpassing many ambitious communities in material interests and in population. The ordinary and even the extraordinary ailments and accidents which pertain to the history of populous towns are encountered among its inhabitants, and in order to meet the demands incident to a voyage of one or two weeks, a regular medical service is provided by the transportation companies.

It has occurred to me that your readers might feel a general interest, some of them, perhaps a special interest, in this department of a great liner's equipment, and that the information which I now have the opportunity to procure may be of practical value to those who, for any reason, may be attracted to this field.

All passenger ships carry one, usually two medical officers—a senior and a junior surgeon. These positions, as a rule, are filled by young physicians who seek these appointments for the purpose of acquiring knowledge and of seeing something of the world before settling down to steady work, although in some instances, men of mature age, but of indolent temperament and with a special fondness for the sea obtain these places and remain in the service for years. It is not infrequent that an established practitioner desirous of a sea trip for health, recreation or pleasure contracts with the ship's company to serve for one or more trips.

The pleasures of a voyage, the opportunity afforded to observe and of recuperation or escape from wearing monotony and exhaustand of recuperation or escape from wearing monotony and exhausting application make these places very attractive and afford the desired advantages without actual expense to the individual.

The salary varies from fifty to seventy-five dollars per month, and in addition to the cash compensation, board, room and offices on the ship are furnished. Besides, the usual fees may be charged the cabin passengers for any service rendered to them. The work, both medical and surgical, is light and varied, and there is little or no drudgery connected with its pursuit. To facilitate the work of the surgeon and to contribute to the comfort of the patients, the boats are provided with hospital wards. These are of two classes. One is set apart for cabin passengers and the other for the steerage. They are similar to like wards in any well ordered hospital or infirmary.

Among the diseases contracted at sea, pneumonia stands preeminent. It is met with among all classes and conditions, and at all seasons, though it is more frequent in winter. The importance of warm clothing and of sufficient covering cannot be overrated, and all prospective travelers unfamiliar with sea travel should be forewarned on this point.

The acute contagious diseases are greatly dreaded. It is especially among the steerage that they are apt to prevail—indeed, they are almost exclusively confined to this class of passengers. Measles, small-pox and scarlet fever are most frequently encountered, and in the order named.

In my next letter I shall endeavor to give you an interesting report from some of the famous hospitals of the old world, as it is my purpose to visit representative institutions in England, France and Germany before my return.

Yours truly,

James B. Baird, Jr.

---

The health officer of Battersea in his annual report declares that inhabitants of London pay at least $150,000 for water added to milk.

